# Monitoring Serum Bisoprolol Concentrations in Patients With Heart Failure With Reduced Ejection Fraction: Results of a Pilot Study From Routine Health Care

**DOI:** 10.1002/prp2.70089

**Published:** 2025-04-26

**Authors:** Ivana Kacirova, Marie Lazarova, Romana Urinovska, Jozef Dodulik, Diana Drienikova, Jan Vaclavik

**Affiliations:** ^1^ Department of Clinical Pharmacology Institute of Laboratory Medicine, University Hospital Ostrava Ostrava Czech Republic; ^2^ Department of Clinical Pharmacology Faculty of Medicine, University of Ostrava Ostrava Czech Republic; ^3^ Department of Internal Medicine and Cardiology University Hospital Ostrava Ostrava Czech Republic; ^4^ Department of Internal Medicine Faculty of Medicine, University of Ostrava Ostrava Czech Republic

**Keywords:** bisoprolol, concentrations, heart failure, variability

## Abstract

Bisoprolol is a second‐generation, highly selective beta‐1 adrenergic receptor antagonist with various beneficial effects in patients with heart failure. Interindividual variability in response to bisoprolol is known, and finding the optimal dose for individual patients with heart failure is still challenging. This pilot study included patients treated with bisoprolol for chronic heart failure with reduced ejection fraction. Between November 2022 and November 2023, one to six blood samples were collected from these patients to determine the trough serum concentration of bisoprolol. At the same time, the values of selected clinical variables were recorded. Bisoprolol concentrations ranged from 1.1 to 65.0 μg/L and correlated with both the daily dose and the dose per kilogram of body weight. However, wide variability in measured serum concentrations of bisoprolol was observed at the same daily dose and in apparent weight‐adjusted clearance. Patients classified as NYHA III–IV received a 33% higher dose per kilogram of body weight than patients in NYHA I–II but achieved 165% higher serum concentrations of bisoprolol. An inverse correlation was found between diastolic blood pressure and dose per kilogram of body weight, and a positive correlation between N‐terminal pro‐B‐type natriuretic peptide and both dose per kilogram of body weight and serum bisoprolol concentration. A wide variability in patients' serum concentrations of bisoprolol achieved after taking the same dose has been observed. A significantly higher concentration‐to‐dose ratio and a significantly lower weight‐adjusted apparent clearance were demonstrated in patients with reduced cardiac function, reduced renal function, and taking the combination with amiodarone. These patients may be more prone to overdose with bisoprolol.

## Graphical abstract

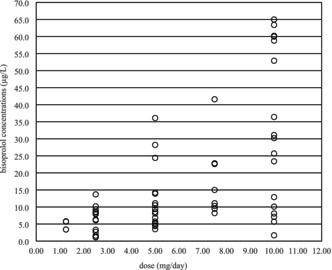

**Abbreviations**
AMI: amiodarone
c/D ratio: concentration‐to‐dose ratio
CL/F: weight‐adjusted apparent clearance
CYP: cytochrome P450
DBP: diastolic blood pressure
eGFR: estimated glomerular filtration rate
HF: heart failure
HFrEF: reduced ejection fraction
HR: heart rate
IMs: intermediate metabolizers
LLoQ: lower limit of quantification
LVEF: left ventricular ejection fraction
NMs: normal metabolizers
NT‐proBNP: N‐terminal pro‐B‐type natriuretic peptide
NYHA: New York Heart Association system
PMs: poor metabolizers
SBP: systolic blood pressure
UMs: ultra‐rapid metabolizers

## 1 Introduction

Bisoprolol acts as a highly selective second‐generation beta‐1 adrenergic receptor antagonist with low affinity for beta‐2 adrenergic receptors. About 50% of it is metabolized by cytochrome P450 (CYP) 2D6 and 3A4 enzymes, and the remainder is excreted unchanged by the kidneys [1]. Due to this balanced elimination, bisoprolol is considered a safer beta‐blocker in patients with impaired liver or kidney function, with a low risk of accumulation and thus a reduced risk of adverse effects [2].

Bisoprolol has been shown to have various beneficial effects in patients with heart failure (HF). The CIBIS II study showed significant reductions in morbidity and mortality in patients with HF with reduced ejection fraction (HFrEF) under treatment with this drug [3]. However, interindividual variability in response to bisoprolol, despite the use of tailored dosage to achieve the goal of blood pressure and heart rate, has been demonstrated in previous studies [4].

Finding the optimal dosage of bisoprolol for individual HF patients is still challenging. To date, little information has been reported on the relationship between plasma concentrations of bisoprolol and HF compensation in real clinical practice. So far, the only study monitoring the distribution of plasma concentration of bisoprolol and its relationship to the worsening of HF was conducted by Arita et al. [5, 6], who found that high plasma bisoprolol concentration was associated with worsening HF in elderly patients and those with HF with preserved ejection fraction.

However, to the best of our knowledge, more detailed information regarding the routine monitoring of serum bisoprolol concentrations in patients with HFrEF has not been published. In our pilot study, serum bisoprolol concentrations were measured in samples obtained during routine health care of patients with HFrEF.

## 2 Method

This pilot study included patients treated with bisoprolol (dosage shown in Table 1) for chronic heart failure with reduced ejection fraction in the Cardiology Outpatient Clinic of the University Hospital Ostrava, Czech Republic. Thirty‐five subjects (9 women, 26 men) with an average age of 57 ± 15 years, weight of 95 ± 27 kg, and height of 177 ± 11 cm were monitored. Between November 2022 and November 2023, one to six blood samples were taken from these patients to determine the serum concentration of bisoprolol. The values of the left ventricular ejection fraction (LVEF), systolic blood pressure (SBP), diastolic blood pressure (DBP), heart rate (HR), N‐terminal pro‐B‐type natriuretic peptide (NT‐proBNP), and the New York Heart Association (NYHA) system value were recorded for each patient. The subjects were instructed to take the morning dose of the drug only after the blood collection. Trough (minimal) serum concentrations were determined by high‐performance liquid chromatography at the Department of Clinical Pharmacology, Institute of Laboratory Medicine, University Hospital Ostrava, Czech Republic, with the lower limit of quantification (LLoQ) being 1.0 μg/L. At the time of bisoprolol concentration sampling, liver enzyme values (alanine aminotransferase, aspartate aminotransferase) and renal function markers (serum creatinine concentration, serum urea concentration, and estimated glomerular filtration rate—eGFR) were determined by a local biochemical laboratory. Other medications used were also recorded. Subsequently, patients were divided into two subgroups according to the values of eGFR (below and equal/above 1 mL/s/1.73 m^2^; reference range = 1.00–2.35 mL/s/1.73 m^2^). The study was reviewed and approved by the local ethics committee (Reference number 379/2024, Ethics Committee of the University Hospital Ostrava, Czech Republic).

**TABLE 1:** Differences in daily dose, dose per kilogram of body weight, serum concentrations, concentrations‐to‐dose (c/D) ratio, and weight‐adjusted apparent clearance (CL/F) of bisoprolol depending on NYHA stage (NYHA I–II vs. NYHA III–IV), concomitant use of amiodarone (AMI; with AMI vs. without AMI), and estimated glomerular filtration rate (eGFR ≥ 1 vs. eGFR < 1; in mL/s/1.73 m^2^); ns = not significant.

	Dose (mg/day)	Dose (mg/day)	Dose/kg (mg/kg)	Dose/kg (mg/kg)	Concentrations (μg/L)	Concentrations (μg/L)	c/D ratio	c/D ratio	CL/F (L/kg)	CL/F (L/kg)
NYHA	I–II	III–IV	I–II	III–IV	I–II	III–IV	I–II	III–IV	I–II	III–IV
Number	43	15	43	15	43	15	43	15	43	15
Median	5.00	7.5	0.06	0.08	8.6	*22.8	1.9	*3.2	4.90	*3.43
Range	1.25–10.00	1.25–10.0	0.01–0.13	0.01–0.13	1.1–63.4	5.2–65.0	0.2–6.3	1.0–7.2	1.47–74.46	1.85–10.80
Mean ± SD	5.38 ± 2.91	6.75 ± 3.33	0.06 ± 0.03	*0.08 ± 0.04	11.9 ± 12.6	28.7 ± 23.3	2.3 ± 1.6	4.0 ± 2.1	8.80 ± 11.31	4.20 ± 2.94
*NYHA I–II × III–IV		ns		*p = 0.0159		*p = 0.0153		*p = 0.0038		*p = 0.0165
Amiodarone	Without AMI	With AMI	Without AMI	With AMI	Without AMI	With AMI	Without AMI	With AMI	Without AMI	With AMI
Number	41	17	41	17	41	17	41	17	41	17
Median	5.0	7.5	0.05	0.08	8.1	*22.8	2.1	*4.6	6.51	*2.55
Range	1.25–10.0	1.25–10.0	0.01–0.13	0.01–0.12	1.1–31.1	3.5–65.0	0.2–5.6	0.7–7.2	1.91–74.46	1.47–17.86
Mean ± SD	5.09 ± 2.98	*7.28 ± 2.73	0.06 ± 0.03	0.07 ± 0.03	10.0 ± 8.0	31.2 ± 24.3	2.2 ± 1.4	4.0 ± 2.3	9.07 ± 11.37	4.10 ± 4.03
*With × without AMI		*p = 0.0117		ns		*p = 0.0010		*p = 0.0047		*p = 0.0007
eGFR	eGFR ≥ 1	eGFR < 1	eGFR ≥ 1	eGFR < 1	eGFR ≥ 1	eGFR < 1	eGFR ≥ 1	eGFR < 1	eGFR ≥ 1	eGFR < 1
Number	34	24	34	24	34	24	34	24	34	24
Median	5.0	5.0	0.05	0.07	8.2	*13.8	1.4	*3.4	7.91	*3.59
Range	1.25–10.0	1.25–10	0.01–0.13	0.01–0.12	1.1–28.2	3.5–65.0	0.2–5.6	0.7–7.2	1.91–74.46	1.47–17.86
Mean ± SD	5.55 ± 2.92	5.99 ± 3.28	0.05 ± 0.03	0.07 ± 0.03	9.2 ± 7.1	26.1 ± 22.6	1.9 ± 1.3	3.9 ± 1.9	9.69 ± 12.37	4.66 ± 3.78
*eGFR ≥ 1 × eGFR < 1		ns		ns		*p = 0.0036		*p < 0.0001		*p = 0.0046

*Note:* * indicates whether the mean or median was used for comparison.

In 3 patients (including one patient in two cases) bisoprolol concentrations were lower than the LLoQ. Those patients were considered non‐adherent, and those concentrations were excluded from further statistical analysis. Weight‐adjusted apparent clearance (CL/F) was calculated for bisoprolol as follows: CL/F (L/kg) = daily dose (mg/kg)/minimal serum concentration (mg/L) [7]. Statistical analysis was performed using GraphPad Prism version 5.00 for Windows (GraphPad Software, San Diego, CA, USA). D'Agostino and Pearson's omnibus normality tests were used to determine whether the results formed a Gaussian distribution. An unpaired t‐test (if values followed a Gaussian distribution) or a non‐parametric Mann–Whitney test was used to compare the distribution of two unpaired groups, and a Pearson or Spearman's nonparametric correlation test was conducted for correlation analysis. A value of *p <* 0.05 was considered statistically significant.

## 3 Results

**FIGURE 1:**
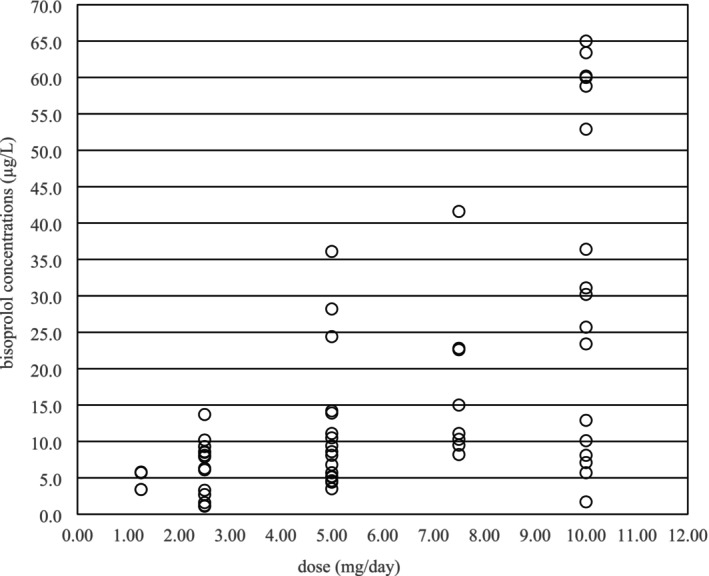
Distribution of bisoprolol serum concentrations at different daily doses.

Bisoprolol concentrations ranged from 1.1 to 65.0 μg/L (median 9.3 μg/L) and correlated with both daily dose (*p* < 0.0001) and dose per kilogram of body weight (*p* < 0.0001). On the other hand, a wide variability was observed in the measured serum concentrations of bisoprolol at the same daily dose (Figure 1) and also in the weight‐adjusted apparent clearance (median = 4.64 L/kg, range = 1.47–74.46 L/kg).

Alanine aminotransferase ranged between 0.15 and 1.75 μkat/L (median 0.44 μkat/L) and aspartate aminotransferase between 0.13 and 0.95 μkat/L (median 0.39 μkat/L). Thus, no patient showed significantly impaired liver function. Serum creatinine concentrations ranged between 59 and 248 μmoL/L (median 96 μmoL/L), serum urea concentrations between 2.3 and 20.6 mmoL/L (median 6.7 mmoL/L), and eGFR between 0.35 and 1.94 mL/s/1.73 m^2^ (median 1.23 mL/s/1.73 m^2^). No concomitant medication affecting bisoprolol‐metabolizing enzymes was reported in 41 samples, while amiodarone (CYP2D6 inhibitor) was reported as concomitant medication in 17 samples [7, 8, 9, 10].

Table 1 shows the differences in daily dose, dose per kilogram of body weight, serum concentrations, c/D ratio, and CL/F of bisoprolol depending on NYHA stage (NYHA I–II vs. NYHA III–IV), concomitant use of amiodarone (with amiodarone vs. without amiodarone), and eGFR values (eGFR ≥ 1 vs. eGFR < 1 mL/s/1.73 m^2^). Patients in NYHA III–IV used a 33% higher dose per kilogram of body weight (*p* = 0.0159) than patients in NYHA I–II, but achieved a 165% higher serum concentration of bisoprolol (*p* = 0.0153), a higher c/D ratio (*p* = 0.0038), and a lower CL/F (*p* = 0.0165). The c/D ratio was significantly higher and CL/F significantly lower also when bisoprolol was administered concomitantly with amiodarone (*p* = 0.0047 for c/D ratio; *p* = 0.0007 for CL/F) or in the presence of reduced renal function (*p* < 0.0001 for c/D ratio; *p* = 0.0046 for CL/F). The largest difference was observed in patients with NYHA III–IV, eGFR < 1 mL/s/1.73 m^2^, concomitantly taking amiodarone compared to patients with NYHA I–II, eGFR ≥ 1 mL/s/1.73 m^2^, not taking amiodarone. In this case, the c/D ratio was 362% higher (*p* < 0.0001) and CL/F was 77% lower (*p* = 0.0002) in patients with NYHA III–IV, reduced renal function, with amiodarone compared to patients with NYHA I–II, normal renal function, without amiodarone (Table 2). Additionally, higher NT‐proBNP values were observed in patients with NYHA III–IV, reduced renal function, with amiodarone compared to patients with NYHA I–II, normal renal function, without amiodarone (*p* = 0.0020).

**TABLE 2:** Differences in daily dose, dose per kilogram of body weight, serum concentrations, concentrations‐to‐dose (c/D) ratio, and weight‐adjusted apparent clearance (CL/F) of bisoprolol and N‐terminal pro‐B‐type natriuretic peptide (NT‐proBNP) depending on NYHA stage, concomitant use of amiodarone (AMI), and estimated glomerular filtration rate (eGFR, in mL/s/1.73 m^2^); *NYHA I–II, without AMI, eGFR ≥ 1 versus NYHA III–IV, with AMI, eGFR < 1; ns = not significant.

		NYHA I–II, without AMI, eGFR ≥ 1 number = 27	NYHA III–IV, with AMI, eGFR < 1 number = 8	NYHA I–II, without AMI, eGFR ≥ 1 versus NYHA III–IV, with AMI, eGFR < 1
Dose (mg/day)	Median (range) Mean ± SD	5.0 (1.25–10.0) 5.32 ± 2.95	8.75 (1.25–10.0) 7.66 ± 3.17	ns
Dose/kg (mg/kg)	Median (range) Mean ± SD	0.06 (0.01–0.13) 0.05 ± 0.03	*0.10 (0.01–0.12) 0.09 ± 0.04	*p = 0.0140
Concentrations (μg/L)	Median (range) Mean ± SD	5.7 (1.1–28.2) 8.9 ± 7.8	*50.2 (5.7–65.0) 43.8 ± 21.3	*p = 0.0005
c/D ratio	Median (range) Mean ± SD	1.3 (0.2–5.6) 1.8 ± 1.4	*6.0 (3.0–7.2) 5.6 ± 1.3	*p < 0.0001
CL/F (L/kg)	Median (range) Mean ± SD	8.88 (1.91–74.46) 10.90 ± 13.62	*2.03 (1.85–3.43) 2.24 ± 0.53	*p = 0.0002
NT‐proBNP (ng/L)	Median (range) Mean ± SD	386 (51–4611) 725 ± 1082	*11587 (373–17 524) 9319 ± 7262	*p = 0.0020

**FIGURE 2:**
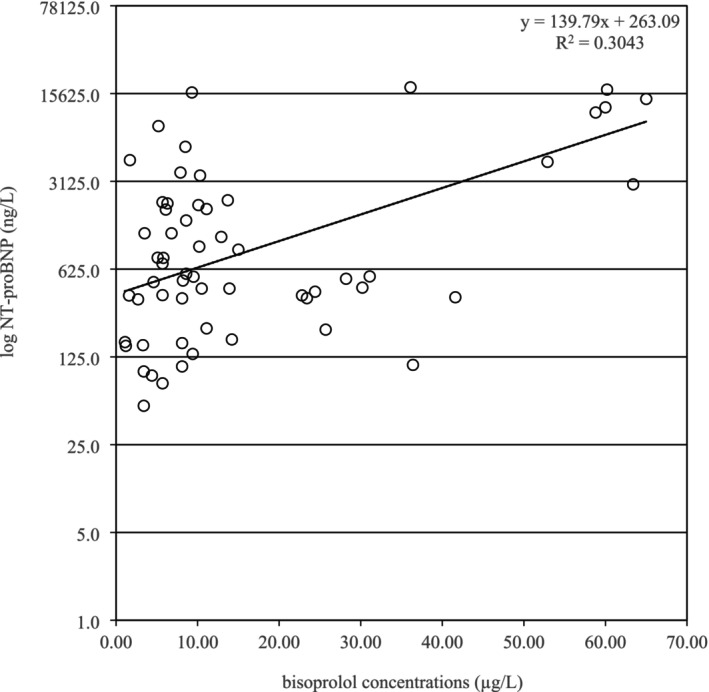
Correlation between serum concentration of bisoprolol and N‐terminal pro‐B‐type natriuretic peptide (NT‐proBNP) in a logarithmic scale.

When analyzing for correlations between daily dose, dose per kilogram of body weight, and serum concentration of bisoprolol and the values of LVEF, SBP, DBP, and HR, an inverse correlation was found between DBP and dose per kilogram of body weight (*p* = 0.0399). On the other hand, there was a positive correlation between NT‐proBNP and both the dose per kilogram of body weight (*p* = 0.0227) and the serum concentration of bisoprolol (*p* = 0.0091; Figure 2).

## 4 Discussion

In this pilot study, serum concentrations of bisoprolol obtained during routine health care for patients with HFrEF and their relationship to selected clinical variables are presented. A significant correlation between the serum concentration of bisoprolol and the daily dose was demonstrated, as in the work of Arita et al. [5]. In addition, a significant correlation was also found between serum bisoprolol concentration and dose per kilogram of body weight. On the other hand, our study demonstrated wide interindividual variability in both serum bisoprolol concentrations achieved with the same daily dose and in weight‐adjusted apparent clearance of bisoprolol. Previous studies have also suggested that bisoprolol use often results in variable responses between patients [4]. Many factors can alter bisoprolol pharmacokinetics, such as reduced renal blood flow, hepatic blood flow, and mass, in addition to the activities of drug metabolizing enzymes (CYP2D6 and CYP3A4) and the influence of genetic polymorphisms [4, 11, 12, 13].

Trobec et al. [14] studied the pharmacokinetics of bisoprolol in 46 patients with HF (NYHA class I/II/III = 2/36/8) and found that the mean clearance of bisoprolol in HF patients was 10.2 L/h, which was about one‐third less than in the healthy population [1, 15]. Nikolic et al. [16] reported a similar clearance of bisoprolol in HF patients (11.4 L/h). The reduced clearance of bisoprolol in HF patients may therefore lead to higher average plasma concentrations compared to the healthy population. Trobec et al. [14] also reported that the clearance of bisoprolol is significantly related to the renal function of the patients. A decrease in renal function may lead to an increase in the average concentration of bisoprolol with a consequent risk of bradycardia, which may require dosage adjustment. Similarly, according to Arita et al. [5, 6], the discrepancy between the dose of bisoprolol and bisoprolol plasma concentration could be explained by age and mainly renal dysfunction. Momčilović et al. [17] demonstrated in hypertensive patients with type 2 diabetes mellitus that the pharmacokinetics of bisoprolol are influenced by creatinine clearance. For this reason, renal function should be examined in these patients before starting bisoprolol treatment, and the dose should be adjusted subsequently to avoid possible accumulation of bisoprolol and its adverse effects [17].

In the case of CYP2D6, genomic variation has been shown to influence the pharmacokinetics and pharmacodynamics of a number of drugs, including another beta1‐specific antagonist, metoprolol [18]. Standardization of the CYP2D6 genotype–phenotype association has established four phenotypic groups in relation to metoprolol metabolism: ultra‐rapid metabolizers (UMs), normal metabolizers (NMs), intermediate metabolizers (IMs), and poor metabolizers (PMs). CYP2D6 PMs show no enzymatic activity, and patients in this phenotype group are expected to have a several‐fold increase in metoprolol concentrations compared to NMs [19, 20, 21, 22]. In contrast, there is a lack of studies on the influence of genetic polymorphisms on the response to other beta‐blockers (bisoprolol, atenolol, carvedilol, etc.). Specifically, for bisoprolol, the results of studies investigating the influence of genetic polymorphisms (especially CYP3A4, CYP2D6, and beta‐adrenergic receptors) on patient responses to treatment are inconclusive [20, 23]. According to a recently published study by Okdy et al. [13], there may be a possible association between the CYP3A5*3, CYP2D6*4, and CYP2D6*2A variants and peak bisoprolol concentrations. On the other hand, a patient's predicted drug metabolism based on genotype can sometimes vary from the true phenotypic capacity, a condition referred to as phenoconversion [20]. One cause of phenoconversion is drug–drug interaction resulting from inhibition of CYP2D6. In this scenario, strong CYP2D6 inhibitors reduce the enzyme's activity to near zero, causing individuals with normal or intermediate genotypes to mimic a PM [24, 25, 26]. Commonly prescribed drugs that are known strong inhibitors of the CYP2D6 enzyme include bupropion, fluoxetine, paroxetine, quinidine, and terbinafine [27]. Amiodarone, and especially its active metabolite desethylamiodarone, act as inhibitors of several CYPs, including CYP2D6 [28, 29]. Co‐administration of amiodarone with drugs metabolized by CYP2D6 (e.g., metoprolol) may therefore lead to increased concentrations of these drugs [30, 31, 32]. Patients taking the combination of metoprolol and amiodarone are at increased risk of bradycardia and atrioventricular block. This is due to the increased concentrations of metoprolol due to inhibition of CYP2D6 by amiodarone and the fact that both amiodarone and metoprolol have beta‐adrenergic blocking properties (i.e., a decrease in heart rate), and amiodarone also has potassium channel blocking properties (negative inotropy) [8, 11, 33, 34]. Duricova et al. [35] presented a case of metoprolol‐propafenone interaction, where high concentrations of metoprolol affected the patient's clinical condition. A patient receiving concomitant metoprolol and propafenone (CYP2D6 inhibitor) was diagnosed as having global heart failure with atrial fibrillation with slow ventricular response. Serum concentrations of metoprolol and its metabolite alpha‐hydroxymetoprolol were measured, and CYP2D6 genotyping was performed. The patient had the IM genotype; however, 3 h after dosing, the metabolic ratio (i.e., the ratio of the parent substance metoprolol and its metabolite alpha‐hydroxymetoprolol) indicated the PM phenotype. After reducing the dose of metoprolol and discontinuing propafenone, the patient's condition improved, and a significant decrease in the metabolic ratio was also evident. This case report is a clear example of phenoconversion, where the patient with the IM genotype converted to the PM phenotype after the addition of propafenone [35]. Serum concentrations of metoprolol and alpha‐hydroxymetoprolol were also measured in our previous pediatric case of death [36] due to a mixed drug overdose of metoprolol and propafenone. The toxicity of metoprolol was potentiated by a drug interaction with propafenone causing inhibition of CYP2D6, resulting in a PM phenotype (pharmacokinetic interaction), as well as a simultaneous negative inotropic effect due to beta‐adrenergic and sodium channel blocking activity (pharmacodynamic interaction) [36]. For bisoprolol, Déniel et al. [37] reported the case of an 82‐year‐old woman treated with bisoprolol 10 mg/day and verapamil 160 mg/day who was admitted for exacerbation of dyspnea and symptoms of congestive heart failure. Blood laboratory values were as follows: creatinine 87 μmol/L (normal 40–100 μmol/L), creatinine clearance 54 mL/min per 1.73 m^2^ (normal 90–140 mL/min per 1.73 m^2^). After initial improvement, on day 3, severe sepsis originating from the urinary tract occurred, with an increase in creatinine level to 179 μmol/L and a decrease in creatinine clearance to 24 mL/min/1.73 m^2^. Toxicological analysis showed a bisoprolol level of 173 ng/mL (therapeutic range 10–60 ng/mL) and a verapamil level of 278 ng/mL (therapeutic range 20–350 ng/mL). The patient experienced cardiac arrest with asystole and ineffective conventional cardiac resuscitation including adrenaline. Déniel et al. reported that the combination of a beta‐blocker and a non‐dihydropyridine calcium channel antagonist should be administered only rarely because of the high risk of severe bradycardia or complete atrioventricular block. In addition, verapamil may increase serum concentrations of beta‐blockers by inhibiting their hepatic metabolism [37]. Hashiyada et al. [38] reported the case of an elderly woman who died of uncontrolled bradycardia in the hospital. The physician prescribed 1.35 mg of bisoprolol orally, but the nurse mistakenly administered 10 mg of the drug to the patient 9 h before her death. Bisoprolol was measured in the blood at a concentration of 176 ng/mL by liquid chromatography‐mass spectrometry. This concentration was twice the expected value, even though the patient had HF. However, the patient was found to have a CYP2D6 mutation that reduces the enzyme activity by half in intermediate metabolizers. In the presented case, the unexplained high bisoprolol blood concentration was probably only partly due to a prescription error [38].

Since no patient in our cohort showed significantly elevated liver enzymes, the observed interindividual variability in bisoprolol pharmacokinetics was not due to liver dysfunction. On the other hand, patients in NYHA III–IV were treated with a 33% higher dose per kilogram of body weight than those in NYHA I–II, but achieved a 165% higher bisoprolol serum concentration, a significantly higher c/D ratio, and a significantly lower CL/F. The c/D ratio was significantly higher, and CL/F was significantly lower also when bisoprolol was administered concomitantly with amiodarone or in the presence of reduced renal function. The largest difference was observed in patients with NYHA III–IV, eGFR < 1 mL/s/1.73 m^2^ and concomitantly taking amiodarone compared to patients with NYHA I–II, eGFR ≥ 1 mL/s/1.73 m^2^, not taking amiodarone. In this case, the c/D ratio was 362% higher, and CL/F was 77% lower in patients with NYHA III–IV, eGFR < 1 mL/s/1.73 m^2^, and amiodarone comedication. Additionally, higher NT‐proBNP values were observed in patients with NYHA III–IV, reduced renal function with amiodarone compared to patients with NYHA I–II, normal renal function without amiodarone.

When analyzing the correlation between daily dose, dose per kilogram of body weight, and serum concentration of bisoprolol and the values of LVEF, SBP, DBP, and HR, only an inverse correlation was found between DBP and dose per kilogram of body weight. In comparison, meta‐regression analyses of beta‐blocker heart failure trials by McAlister et al. [39] demonstrated that the magnitude of survival benefit seen with beta‐blockers was statistically significantly associated with the magnitude of heart rate reduction achieved, but not the dosage of beta‐blocker administered. A key feature of the pathophysiology of heart failure is excessive sympathetic nervous system activation, which leads to adverse remodeling of the left ventricular wall and thus contributes to HF. Recent views suggest that treatment of HF should not be guided solely by LVEF but should also consider signs and symptoms of HF (e.g., edema and tachycardia), the severity of HF, and comorbid conditions. Beta‐blockers improve symptoms and functional status in patients with HF by reducing the activity of the renin‐angiotensin‐aldosterone system. These drugs have been shown in randomized, placebo‐controlled trials to improve survival and also reduce the risk of other important clinical outcomes, such as hospitalization for HF. Higher doses of beta‐blockers have also been shown to be associated with better clinical outcomes in patients with HF, so it is important to ensure adequate titration of therapy to their maximum (or maximally tolerated) doses to ensure optimal outcomes [40]. On the other hand, we observed a positive correlation between NT‐proBNP and both the dose per kilogram of body weight and the serum concentration of bisoprolol. Similarly, Arita et al. [5], who analyzed trough plasma concentrations of bisoprolol in their single‐center observational study in 114 patients with HF, found that high plasma concentrations of bisoprolol could be associated with worsening of HF. In view of the negative inotropic and chronotropic effects of bisoprolol, caution may be necessary in some patients with HF in regard to the risk of overdose with this beta‐blocker. Expecting beneficial effects on HF, treating physicians may tend to use higher doses of bisoprolol, which may actually worsen HF. Greater attention should be paid in particular to elderly patients and patients with impaired renal function, as there may be a discrepancy between the dose of bisoprolol and its pharmacokinetics and consequently its pharmacodynamic effect [5].

Therefore, it would be appropriate to conduct a prospective longitudinal study to monitor the relationship between serum bisoprolol concentrations and therapeutic/adverse effects. Based on the results obtained, it would be possible to evaluate whether the dose of bisoprolol or its serum concentration better correlates with the development of clinical and paraclinical parameters or with the prognosis of patients with HFrEF. If this hypothesis were confirmed, the introduction of a method of therapeutic monitoring of bisoprolol concentrations in patients with HFrEF could serve as an effective tool to detect changes in bisoprolol pharmacokinetics, objectify drug interactions, and monitor patient adherence to treatment.

## 5 Limitations

There are several limitations to this project. This is a pilot cross‐sectional study, where an unequal number of blood samples were taken from patients to determine the serum concentration of bisoprolol, and it is therefore not possible to evaluate the long‐term relationship between the determined concentrations and selected clinical variables. Data obtained during routine ambulatory care of patients with HFrEF were analyzed, and the correctness of this information cannot therefore be completely relied upon. In particular, it was not possible to guarantee that the sample was collected before the morning dose of the drug or that the patients adhered fully to the treatment.

However, as this is, to the best of our knowledge, the first prospective study monitoring serum concentrations of bisoprolol in patients with HFrEF, we believe that the findings could be important both for the treatment of patients with HFrEF and for future research and personalization of pharmacotherapy in patients with this chronic disease.

## 6 Conclusions

In this pilot cross‐sectional study, serum concentrations of bisoprolol obtained during routine health care of patients with HFrEF were measured, and their relationship to selected clinical variables was analyzed. A significant correlation was demonstrated between the serum concentration of bisoprolol and both the daily dose and dose per kilogram of body weight. However, wide interindividual variability was observed in the serum concentrations of bisoprolol achieved at the same daily dose and in the weight‐adjusted apparent clearance, and these were related to NYHA stage, combination with amiodarone, and reduced renal function. Our results support the claim that high serum concentrations of bisoprolol may be associated with worsening HF.

When analyzing possible correlations between daily dose, dose per kilogram of body weight, and serum concentration of bisoprolol and selected clinical variables, only an inverse correlation between DBP and dose per kilogram of body weight was found. In contrast, no correlation was evident in relation to SBP, LVEF, or heart rate.

## Author Contributions

Marie Lazarova, Ivana Kacirova, and Romana Urinovska contributed to the study conception and design. Material preparation, data collection, and analysis were performed by Marie Lazarova, Romana Urinovska, Jozef Dodulik, Diana Drienikova, and Jan Vaclavik. The first draft of the manuscript was written by Ivana Kacirova, and all authors commented on previous versions of the manuscript. All authors read and approved the final manuscript.

## Ethics Statement

The possibility to retrospectively process and publish the obtained data was reviewed and approved by the Ethics Committee of Ostrava University Hospital, Czech Republic (Reference number 379/2024).

## Consent

As the analyzed data were obtained during routine health care, the patients did not sign informed consent.

## Conflicts of Interest

The authors declare no conflicts of interest.

## Data Availability

Data not available/The data that has been used is confidential.

## References

[1] G. Leopold , J. Pabst , W. Ungethum , and K. U. Buhring , “Basic Pharmacokinetics of Bisoprolol, a New Highly Beta 1‐Selective Adrenoceptor Antagonist,” Journal of Clinical Pharmacology 26, no. 8 (1986): 616–621, 10.1002/j.1552-4604.1986.tb02959.x. 2878941

[2] V. Fontana , R. M. Turner , B. Francis , et al., “Chromosomal Region 11p14.1 Is Associated With Pharmacokinetics and Pharmacodynamics of Bisoprolol,” Pharmacogenomics and Personalized Medicine 15 (2022): 249–260, 10.2147/PGPM.S352719. 35356681 PMC8958266

[3] Investigators CI , “The Cardiac Insufficiency Bisoprolol Study II (CIBIS‐II): A Randomised Trial,” Lancet 353, no. 9146 (1999): 9–13. 10023943

[4] J. Shin and J. A. Johnson , “Pharmacogenetics of b‐Blockers,” Pharmacotherapy: The Journal of Human Pharmacology and Drug Therapy 27 (2007): 874–887. 10.1592/phco.27.6.874 PMC2735790 17542770

[5] T. Arita , S. Suzuki , Y. Kato , et al., “Association Between Bisoprolol Plasma Concentration and Worsening of Heart Failure: (CVI ARO 6),” Drug Metabolism and Pharmacokinetics 35, no. 2 (2020): 228–237, 10.1016/j.dmpk.2020.01.002. 32044255

[6] T. Arita , S. Suzuki , Y. Kato , et al., “Association Between Dose and Plasma Concentration of Bisoprolol in Patients With Heart Failure (CVI ARO 6),” International Heart Journal 61, no. 4 (2020): 748–754, 10.1536/ihj.20-052. 32684605

[7] E. L. Johnson , Z. N. Stowe , J. C. Ritchie , et al., “Carbamazepine Clearance and Seizure Stability During Pregnancy,” Epilepsy & Behavior 33 (2014): 49–53, 10.1016/j.yebeh.2014.02.011. 24632353 PMC4040964

[8] J. Leor , D. Levartowsky , C. Sharon , and Z. Farfel , “Amiodarone and Beta‐Adrenergic Blockers: An Interaction With Metoprolol but Not With Atenolol,” American Heart Journal 116, no. 1 Pt 1 (1988): 206–207, 10.1016/0002-8703(88)90275-x. 3394625

[9] D. Werner , H. Wuttke , M. F. Fromm , et al., “Effect of Amiodarone on the Plasma Levels of Metoprolol,” American Journal of Cardiology 94, no. 10 (2004): 1319–1321, 10.1016/j.amjcard.2004.07.125. 15541258

[10] K. Fukumoto , T. Kobayashi , K. Tachibana , et al., “Effect of Amiodarone on the Serum Concentration/Dose Ratio of Metoprolol in Patients With Cardiac Arrhythmia,” Drug Metabolism and Pharmacokinetics 21, no. 6 (2006): 501–505, 10.2133/dmpk.21.501. 17220566

[11] S. Robert , M. O. Pilon , E. Oussaïd , et al., “Impact of Amiodarone Use on Metoprolol Concentrations, α‐OH‐Metoprolol Concentrations, Metoprolol Dosing and Heart Rate: A Cross‐Sectional Study,” Pharmacology Research & Perspectives 11, no. 5 (2023): e01137, 10.1002/prp2.1137. 37732835 PMC10512912

[12] M. Taguchi , T. Nozawa , A. Igawa , et al., “Pharmacokinetic Variability of Routinely Administered Bisoprolol in Middle‐Aged and Elderly Japanese Patients,” Biological & Pharmaceutical Bulletin 28, no. 5 (2005): 876–881, 10.1248/bpb.28.876. 15863897

[13] S. M. Okda , N. A. El‐Bassiouny , A. M. El Amrawy , A. Salahuddin , S. M. Elonsy , and A. B. Kassem , “Impact of CYP2D6*2A, CYP2D6*4 and CYP3A5*3 Genetic Polymorphisms on Bisoprolol Peak Concentration and Clinical Response in Acute Coronary Syndrome Patients,” British Journal of Clinical Pharmacology 90, no. 10 (2024): 2539–2553, 10.1111/bcp.16134. 38886107

[14] K. C. Trobec , I. Grabnar , M. K. Kos , et al., “Bisoprolol Pharmacokinetics and Body Composition in Patients With Chronic Heart Failure: A Longitudinal Study,” European Journal of Clinical Pharmacology 72, no. 7 (2016): 813–822, 10.1007/s00228-016-2041-1. 26996442

[15] W. Kirch , I. Rose , H. G. Demers , G. Leopold , J. Pabst , and E. E. Ohnhaus , “Pharmacokinetics of Bisoprolol During Repeated Oral Administration to Healthy Volunteers and Patients With Kidney or Liver Disease,” Clinical Pharmacokinetics 13 (1987): 110–117. 2887325 10.2165/00003088-198713020-00003

[16] V. N. Nikolic , T. Jevtovic‐Stoimenov , R. Velickovic‐Radovanović , et al., “Population Pharmacokinetics of Bisoprolol in Patients With Chronic Heart Failure,” European Journal of Clinical Pharmacology 69 (2013): 859–865. 23093041 10.1007/s00228-012-1427-y

[17] S. Momčilović , A. Jovanović , D. Radojković , et al., “Population Pharmacokinetic Analysis of Bisoprolol in Type 2 Diabetic Patients With Hypertension,” European Journal of Clinical Pharmacology 76, no. 11 (2020): 1539–1546, 10.1007/s00228-020-02937-6. 32583355

[18] T. Rau , H. Wuttke , L. M. Michels , et al., “Impact of the CYP2D6 Genotype on the Clinical Effects of Metoprolol: A Prospective Longitudinal Study,” Clinical Pharmacology and Therapeutics 85, no. 3 (2009): 269–272, 10.1038/clpt.2008.218. 19037197

[19] Pharmacogene Variation Consortium (PharmVar) , “CYP2D6,” accessed November 20, 2024, https://www.pharmvar.org/gene/CYP2D6.

[20] S. Collett , A. Massmann , N. J. Petry , et al., “Metoprolol and CYP2D6: A Retrospective Cohort Study Evaluating Genotype‐Based Outcomes,” Journal of Personalized Medicine 13, no. 3 (2023): 416, 10.3390/jpm13030416. 36983598 PMC10058912

[21] C. M. Blake , E. D. Kharasch , M. Schwab , and P. Nagele , “A Meta‐Analysis of CYP2D6 Metabolizer Phenotype and Metoprolol. Pharmacokinetics,” Clinical Pharmacology and Therapeutics 94 (2013): 394–399, 10.1038/clpt.2013.96. 23665868 PMC3818912

[22] L. Dean and M. Kane , “Metoprolol Therapy and CYP2D6 Genotype,” April 4, 2017 [Updated September 19, 2024], in Medical Genetics Summaries, [Internet], ed. V. M. Pratt , S. A. Scott , M. Pirmohamed , et al. (National Center for Biotechnology Information (US), 2012), accessed November 20, 2024, https://www.ncbi.nlm.nih.gov/books/NBK425389/.

[23] C. Castaño‐Amores , X. Díaz‐Villamarín , A. M. Pérez‐Gutiérrez , et al., “Pharmacogenetic Polymorphisms Affecting Bisoprolol Response,” Biomedicine & Pharmacotherapy 142 (2021): 112069, 10.1016/j.biopha.2021.112069. 34470728

[24] R. R. Shah and R. L. Smith , “Addressing Phenoconversion: The Achilles' Heel of Personalized Medicine,” British Journal of Clinical Pharmacology 79 (2015): 222–240, 10.1111/bcp.12441. 24913012 PMC4309629

[25] S. D. Klomp , M. L. Manson , H. J. Guchelaar , and J. J. Swen , “Phenoconversion of Cytochrome P450 Metabolism: A Systematic Review,” Journal of Clinical Medicine 9 (2020): 2890, 10.3390/jcm9092890. 32906709 PMC7565093

[26] E. J. Cicali , D. M. Smith , B. Q. Duong , L. G. Kovar , L. H. Cavallari , and J. A. Johnson , “A Scoping Review of the Evidence Behind CYP2D6 Inhibitor Classifications,” Clinical Pharmacology & Therapeutics 108 (2020): 116–125, 10.1002/cpt.1768. 31910286 PMC7292748

[27] Drug Development and Drug Interactions , “Table of Substrates, Inhibitors and Inducers,” accessed November 23, 2024, https://www.fda.gov/drugs/drug‐interactions‐labeling/drug‐development‐and‐drug‐interactions‐table‐substrates‐inhibitors‐and‐inducers.

[28] K. Ohyama , M. Nakajima , M. Suzuki , N. Shimada , H. Yamazaki , and T. Yokoi , “Inhibitory Effects of Amiodarone and Its N‐Deethylated Metabolite on Human Cytochrome P450 Activities: Prediction of In Vivo Drug Interactions,” British Journal of Clinical Pharmacology 49, no. 3 (2000): 244–253, 10.1046/j.1365-2125.2000.00134.x. 10718780 PMC2014912

[29] K. Ohyama , M. Nakajima , S. Nakamura , N. Shimada , H. Yamazaki , and T. Yokoi , “A Significant Role of Human Cytochrome P450 2C8 in Amiodarone N‐Deethylation: An Approach to Predict the Contribution With Relative Activity Factor,” Drug Metabolism and Disposition 28, no. 11 (2000): 1303–1310. 11038157

[30] B. Berger , F. Bachmann , U. Duthaler , S. Krähenbühl , and M. Haschke , “Cytochrome P450 Enzymes Involved in Metoprolol Metabolism and Use of Metoprolol as a CYP2D6 Phenotyping Probe Drug,” Frontiers in Pharmacology 9 (2018): 774, 10.3389/fphar.2018.00774. 30087611 PMC6066528

[31] M. Meloche , M. Khazaka , I. Kassem , A. Barhdadi , M.‐P. Dubé , and S. de Denus , “CYP2D6 Polymorphism and Its Impact on the Clinical Response to Metoprolol: A Systematic Review and Meta‐Analysis,” British Journal of Clinical Pharmacology 86, no. 6 (2020): 1015–1033, 10.1111/bcp.14247. 32090368 PMC7256128

[32] A. Zamir , I. Hussain , A. Ur Rehman , et al., “Clinical Pharmacokinetics of Metoprolol: A Systematic Review,” Clinical Pharmacokinetics 61, no. 8 (2022): 1095–1114, 10.1007/s40262-022-01145-y. 35764772

[33] M. Kawabata , Y. Yokoyama , T. Sasaki , et al., “Severe Iatrogenic Bradycardia Related to the Combined Use of Beta‐Blocking Agents and Sodium Channel Blockers,” Clinical Pharmacology 7 (2015): 29–36, 10.2147/cpaa.S77021. 25733934 PMC4337503

[34] F. Boutitie , J. P. Boissel , S. J. Connolly , et al., “Amiodarone Interaction With β‐Blockers: Analysis of the Merged EMIAT (European Myocardial Infarct Amiodarone Trial) and CAMIAT (Canadian Amiodarone Myocardial Infarction Trial) Databases. The EMIAT and CAMIAT Investigators,” Circulation 99, no. 17 (1999): 2268–2275, 10.1161/01.cir.99.17.2268. 10226092

[35] J. Duricova , I. Perinova , N. Jurckova , I. Kacirova , and M. Grundmann , “Clinically Important Interaction Between Metoprolol and Propafenone,” Canadian Family Physician 59, no. 4 (2013): 373–375. 23585605 PMC3625083

[36] I. Kacirova , M. Grundmann , M. Kolek , E. Vyskocilova‐Hrudikova , R. Urinovska , and P. Handlos , “Lethal Suicide Attempt With a Mixed‐Drug Intoxication of Metoprolol and Propafenone – A First Pediatric Case Report,” Forensic Science International 278 (2017): e34–e40, 10.1016/j.forsciint.2017.06.025. 28716517

[37] A. Déniel , S. Fedrizzi , V. Lelong‐Boulouard , A. Coquerel , and J. Alexandre , “Fatal Cardiac Arrest Associated With Concomitant Bisoprolol and Verapamil Overdose,” Journal of the American Geriatrics Society 64, no. 2 (2016): 451–452, 10.1111/jgs.13972. 26889857

[38] M. Hashiyada , K. Usui , Y. Hayashizaki , et al., “Unexpectedly High Blood Concentration of Bisoprolol After an Incorrect Prescription: A Case Report,” Legal Medicine (Tokyo, Japan) 15, no. 2 (2013): 103–105, 10.1016/j.legalmed.2012.09.004. 23219584

[39] F. A. McAlister , N. Wiebe , J. A. Ezekowitz , A. A. Leung , and P. W. Armstrong , “Meta‐Analysis: Beta‐Blocker Dose, Heart Rate Reduction, and Death in Patients With Heart Failure,” Annals of Internal Medicine 150, no. 11 (2009): 784–794, 10.7326/0003-4819-150-11-200906020-00006. 19487713

[40] M. T. de Oliveira, Jr. , R. Baptista , S. A. Chavez‐Leal , and M. G. Bonatto , “Heart Failure Management With β‐Blockers: Can We Do Better?,” Current Medical Research and Opinion 40, no. sup1 (2024): 43–54, 10.1080/03007995.2024.2318002. 38597068

